# Malignant gastrointestinal neuroectodermal tumor: a case report and review of the literature

**DOI:** 10.1186/s13000-017-0620-9

**Published:** 2017-03-20

**Authors:** Mohammed J. Alyousef, Jumana A. Alratroot, Tarek ElSharkawy, Mohamed A. Shawarby, Mohammad A. Al hamad, Tarek M. Hashem, Ahmed Alsayyah

**Affiliations:** Department of Pathology and Laboratory Medicine, King Fahd Hospital of University, College of Medicine, University of Dammam, Dammam, Saudi Arabia

**Keywords:** Malignant gastrointestinal neuroectodermal tumor, Clear cell sarcoma-like tumor of gastrointestinal tract, EWSR1

## Abstract

**Background:**

Malignant gastrointestinal neuroectodermal tumor (GNET) is an extremely rare entity that was first described by Zambrano et al. in 2003 as “Clear cell sarcoma-like tumor of the gastrointestinal tract”. It shares some of the histological features of clear cell sarcoma (CCS) but lacks the immunohistochemical reactivity for melanocytic markers. We report a case of GNET that was initially misdiagnosed as gastrointestinal stromal tumor (GIST). Recognizing this entity is important to avoid misdiagnosis.

**Case presentation:**

A case of an 18-year-old male presented with a small intestinal tumor. Histologically it was characterized by polygonal cells arranged in pseudoalveolar pattern and situated in the muscularis propria. Scattered osteoclast-like multinucleated giant cells were also noted. The neoplastic cells were positive for S-100 protein and negative for HMB-45, Melan A, smooth muscle actin, desmin and CD117. EWSR1 gene rearrangement was detected by fluorescence in situ hybridization (FISH) analysis. The patient returned with recurrence after 36 months’ management by surgical resection and died one year later.

**Conclusions:**

GNET can be mistaken histologically for other non-epithelial gastrointestinal tumors. Awareness of its existence and diagnostic criteria by the pathologist is necessary to avoid misdiagnosis, particularly as GIST, CCS or malignant peripheral nerve sheath tumor (MPNST).

## Background

Clear cell sarcoma-like tumor of the gastrointestinal tract (CCSLTGT) or as recently designated, malignant gastrointestinal neuroectodermal tumor (GNET) is an extremely rare and controversial entity [1]. It was first described in 2003 by Zambrano et al. who reported 6 cases as malignant mesenchymal neoplasm of gastrointestinal tract that is characterized by the presence of osteoclast-like multinucleated giant cells, histologically, and expresses S100 protein and is negative for CD117 and melanocytic markers, immunohistochemically. The authors concluded that this entity shares some but not all the features of clear cell sarcoma (CCS) [2]. Up to our knowledge, only 44 cases that may represent GNET were described in the English literature, 29 of which reported as GNET or CCSLTGT [1–9] and 15 as CCS in GI but lacked melanocytic differentiation [10–19]. The clinical, morphologic, immunohistochemical and molecular/cytogenetic features of previously reported GNET cases are presented in (Table 1). GNET can be histologically misdiagnosed as gastrointestinal stromal tumor (GIST), CCS or malignant peripheral nerve sheath tumor (MPNST) by a pathologist unaware of its existence.

**Table 1:** Previously reported cases of malignant gastrointestinal neuroectodermal tumor

	Report	Age (years)/ Sex	Location	Morphology	S100	HMB-45 and Melan A	EM	Genetic finding	Outcome	Additional findings
1	Zambrano et al. [2]	15/F	Small intestine	Oval, spindled	+	-	No melanosomes	t(12;22) (q13;q12)	DOD at 16 months	OLGC
2		21/F	Small intestine	Oval, spindled	+	-	ND	ND	DOD at 12 months	OLGC
3		35/F	Small intestine	Oval, spindled	+	-	No melanosomes	ND	LN and liver metastasis at 12 months	OLGC
4		37/F	Small intestine	Oval, spindled	+	-		ND	NA	OLGC
5		13/M	Stomach	Oval, spindled	+	-	No melanosomes	ND	recurrences at 12 months	OLGC
6		32/M	Small intestine	Oval, spindled	+	-	ND	ND	LN mets	OLGC
7	Stockman et al. [1]^a^	30/F	Jejunum	Epithelioid	+	-	Dense-core granules	EWSR1-ATF1	AWD at 21 months	
8		35/M	Jejunum	Epithelioid	+	-	Dense-core granules	EWSR1-ATF1	DOD at 18 months	
9		33/M	Ileum	Epithelioid	+	-	Dense-core granules	EWSR1-CREB1	AWD at 1.5 months	
10		50/F	Stomach	Epithelioid	+	-	Dense-core granules	EWSR1-ATF1	AWD at 24 months	
11		20/F	Small intestine	Spindled	+	-	Dense-core granules	EWSR1	NED at 20 months	
12		52/M	Ileum	Epithelioid	+	-	ND	-	DOD at 22 months	
13		46/M	Stomach	Epithelioid	+	-	ND	EWSR1	NA	
14		34/F	Stomach	Epithelioid, Spindled	+	-	ND	EWSR1-ATF1	DOD at 19 months	
15		37/F	Ileum	Epithelioid	+	ND	ND	ND	NA	
16		77/F	Colon	Epithelioid, Spindled	+	-	ND	EWSR1-ATF1	DOD at 106 months	
17		31/M	Colon	Epithelioid	+	-	ND	-	DOD at 3 months	
18		17/M	Small intestine	Epithelioid	+	-	ND	EWSR1	NA	
19		60/M	Iluem	Epithelioid	+	-	ND	EWSR1-ATF1	AWD at 36 months	
20		60/F	Jejunum	Epithelioid, Spindled	+	-	ND	EWSR1-CREB1	NED at 41 months	
21		56/M	Stomach	Epithelioid	+	-	ND	EWSR1-CREB1	NA	
22		28/F	Small intestine	Epithelioid	+	-	ND	EWSR1	DOD at 23 months	
23	Insabato et al. [6]	29/M	Stomach	Epithelioid, spindled	+	-	No melanosomes	EWSR1	AWD at 74 months	Hx of Ewing sarcoma, FLI-1+
24	Zhao et al. [7]	33/F	Ileum	Epithelioid	+	-		EWSR1	NA	
25	Kong et al. [5]	17/M	Stomach	Spindled, round	+	-	ND	EWSR1	NED at 10 months	OLGC
26	Huang et al. [3]	40/M	Stomach	Spindled, oval	+	-	ND	ND	LN mets at diagnosis	OLGC
27	Thway et al. [8]	33/M	Small intestine	Epithelioid, spindled	+	-	ND	EWSR1-CREB1	DOD at 7 months	Hx of hepato-blastoma
28	Friedrichs et al. [4]	41/M	Small intestine	Epithelioid	+	-	ND	t(12;22) (q13;q12)	Liver mets at 6 months	OLGC
29	Boland et al. [9]	46/F	Stomach	Epithelioid	+	-	ND	EWSR1-ATF1	NA	Oncocytic change

*AWD* alive with disease, *DOD* dead of disease, *EM* electron microscopy, *Hx* history, *mets*, metastasis, *NA* not available, *NED* no evidence of disease, *ND* not done, *OLGC* osteoclast-like giant cells

^a^Eight cases described by Stockman et al. showed osteoclast-like giant cells

## Case presentation

An 18-year-old college student was referred from the University Clinic for having low hemoglobin (Hg 4.7 g/dl). He was completely well till one month back when he presented with easy fatigability, postural dizziness, palpitation and dyspnea. He gave history of 30 kg weight loss over the past 6 months.

On physical examination, he looked pale, not jaundiced or cyanosed. His vital signs were all within normal limits, except for an elevated heart rate (98/min). Cardiovascular, respiratory and abdominal examinations were unremarkable. Primary investigations in our hospital revealed low hemoglobin level (5.1 g/dl), low iron (<10 ug/dl), and a positive occult blood test. A provisional diagnosis of microcytic hypochromic anemia for further workup was made.

Bone marrow aspirate and trephine biopsy revealed normocellular marrow, depicting normal trilineage hematopoiesis. Upper GI endoscopy showed erosive antral gastritis with patchy ulcerative inflammation. Abdominal ultrasound showed a slightly enlarged liver with a rounded echogenic lesion in the anterior wall of the right lobe suggestive of hemangioma. The spleen was slightly enlarged and normal in echogenicity with no focal lesions.

Computed Tomography (CT) scan of the abdomen and pelvis showed a fairly large well defined soft tissue mass located in the anterior upper pelvis and engulfing jejunal loops causing bowel wall thickening. The patient underwent an exploratory laparotomy and excision of the jejunal mass. Macroscopic examination revealed an 11 × 8 × 2 cm annular mass located within the jejunal wall ulcerating through the mucosa and extending to the serosal surface.

**Fig. 1:**
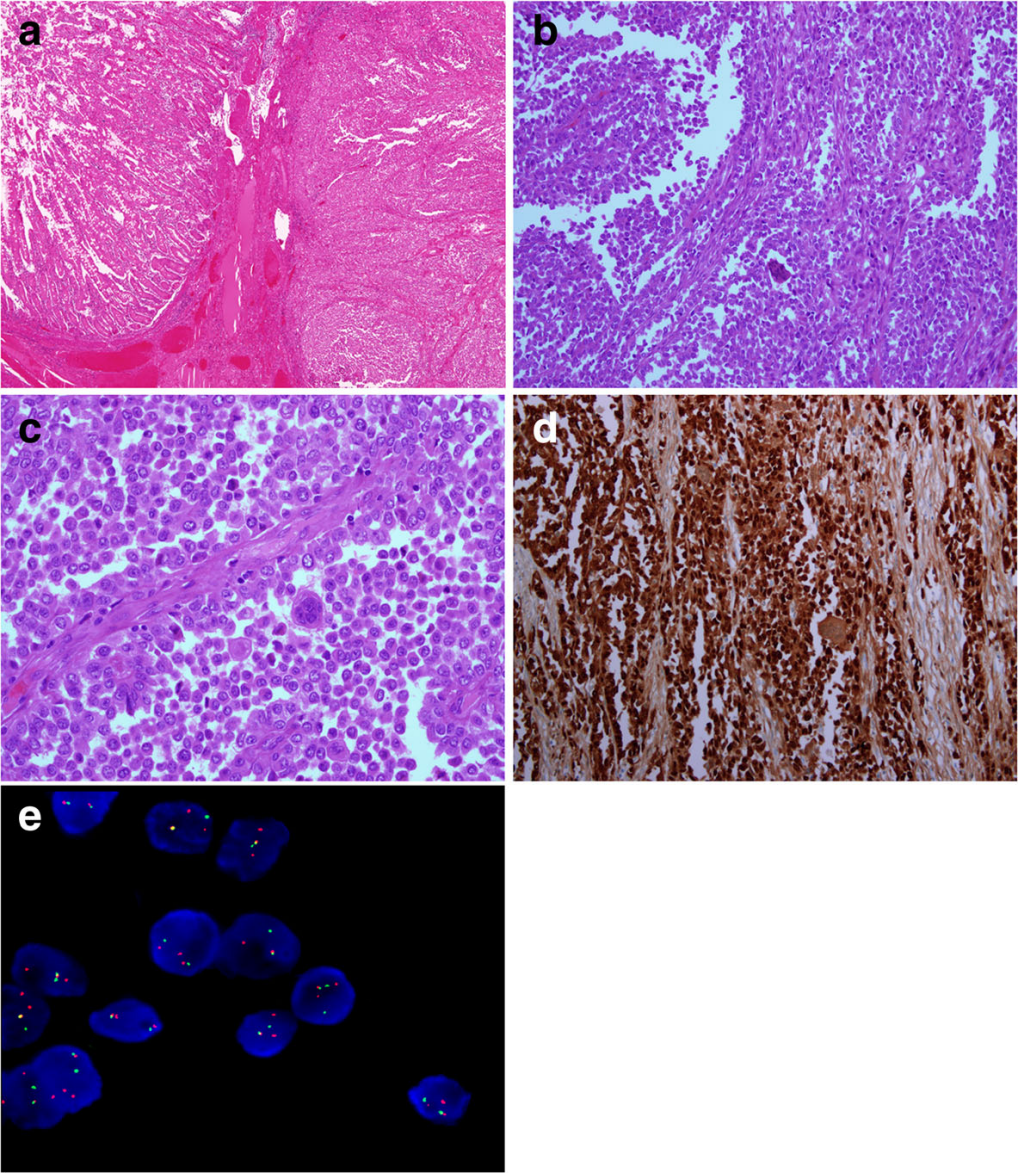
**a** The tumor (right) is extending to the mucosa (Hematoxylin-eosin, original magnification ×40). **b** Tumor cells are arranged in pseudoalveolar pattern. An osteoclast-like giant cell is also present. (Hematoxylin-eosin, original magnification × 200). **c** The cells are polygonal in shape with variable amount of eosinophilic to clear cytoplasm. Characteristic osteoclast-like giant cells are dispersed in between. (Hematoxylin-eosin, original magnification × 400). **d** Strong and diffuse S100 protein staining. (Original magnification × 200). **e** FISH analysis using dual-color, EWSR1 breakapart probe. The tumor cells show one fusion signal pattern, one green and two red, indicative of a rearrangement of one copy of EWSR1 region

Microscopic examination revealed a tumor situated in the muscularis propria and extending to the mucosa and the serosa. The neoplastic cells were arranged in predominantly pseudoalveolar pattern (Fig. 1a,b). They were polygonal in shape with variable amount of eosinophilic to clear cytoplasm (Fig. 1c). The nuclei were oval with vesicular chromatin and inconspicuous nucleoli. Scattered osteoclast-like multinucleated giant cells were also identified (Fig. 1b,c). Frequent mitotic figures and necrosis were noted. All lymph nodes were not involved by tumor. Immunohistochemically, the neoplastic cells were strongly and diffusely positive for S-100 protein (Fig. 1d). They were also positive for vimentin. Melanocytic markers (HMB45 and Melan A), neuroendocrine markers (chromogranin A, synaptophysin and CD56), smooth muscle actin, desmin, CD34, CD117, cytokeratin and LCA were all negative in the neoplastic cells.

Fluorescein in situ hybridization (FISH) analysis for EWSR1 break apart probe on paraffin-embedded tumor showed evidence of a 22q12 rearrangement in 197 out of 205 (96%) of interphase nuclei scored. The native state of EWSR1 break apart probe will be seen as two adjacent or fused (overlapping) red/green (yellow) signals. However, EWSR1 gene rearrangement presented as one red and one green separated signal (Fig. 1e).

The patient did not receive chemotherapy or radiotherapy. During the clinical follow up, the patient remained disease free for 3 years until he presented with local recurrence. The treatment plan was surgical resection but the patient sought medical advice in a different institution where he died of the disease a year later.

## Discussion and Conclusions

GNET tends to occur mainly in young to middle-aged adults. The patients present with abdominal pain, weight loss, intestinal obstruction or an abdominal mass. Some patients have metastatic disease at the time of diagnosis. Most of the cases have been described in the small intestine in addition to the stomach and the colon [1, 3, 5]. The reported cases followed an aggressive clinical behavior with local recurrence and metastatic disease to lymph nodes or hematogenous spread early or at the time of diagnosis [2, 3, 6]. The etiology is unknown. Two case reports documented a GNET that developed after several years’ history of different other primary tumors (Hepatoblastoma and neuroblastoma) that were treated with chemotherapy and radiotherapy suggesting that radiotherapy may play a role in development of these tumor [8, 13]. A case of GNET was reported as a second malignant neoplasm after history of childhood Ewing sarcoma suggesting that they might be related [6].

GNET is centered in the wall of GI tract with extension to the mucosa and/or serosa. The tumor cells grow in solid sheets with pseudopapillary, alveolar and nest formation. The neoplastic cells are predominantly epithelioid with oval or round nuclei with variable amount of eosinophilic or clear cytoplasm but a case featuring an oncocytic cytoplasm has been reported. Fascicles of spindle cells have been also described. The nuclei display irregular nuclear contour. The nucleoli are inconspicuous but occasionally prominent and basophilic. Mitotic activity is variable and ranging from 0 to 20 mitoses per 10 HPF. Necrosis and surface ulceration can be seen. Osteoclast-like multinucleated giant cells is a frequent and consistent finding in most of the tumors. Rosette-like structures and myxoid stroma have been described. Metastatic tumors resemble the primary tumor in morphologic features including the presence of osteoclast-like multinucleated giant cells [1, 2, 5, 7, 9]. Our case occurred in the small intestine and followed an aggressive course of disease with local recurrence and unfortunate death of the patient 4 years after initial presentation. The neoplastic cells showed similar histology to what has been described in literature including the presence of osteoclast-like multinucleated giant cells.

Immunohistochemically, the neoplastic cells are characterized by strong and diffuse staining for S100 protein. They also stain with SOX10. Variable reactivity is described with CD56, synaptophysin and neuron-specific enolase. All cases reported were uniformly negative for HMB-45, Melan-A, tyrosinase and MiTF. They also did not express GIST markers (CD117, DOG-1 and CD34). Desmin and SMA were also negative. Epithelial markers were negative except in one case that expressed CAM 5.2, but only focally and weakly [1, 2]. FLI-1 reactivity was reported to be strongly positive in one patient. This patient was diagnosed and treated of Ewing sarcoma with complete remission 24 years prior to the appearance of GNET. This finding in addition to EWSR1 gene rearrangement raised the issue if Ewing sarcoma is related to GNET [6]. Our case showed strong and diffuse S100 protein reactivity and was negative for CD117, HMB-45 and melan-A. We were not aware of GNET at that time and GIST diagnosis was made after exclusion of other mesenchymal and epithelial tumors at that location.

Of the few cases examined ultrastructurally, no melanocytic, myoid or any other specific differentiation was revealed. Instead, the neoplastic cells showed slender or bulbous interdigitating cell processes in addition to dense-core secretory granules [1, 2].

Antonescu and his team studied 3 cases of CCS of gastrointestinal tract and claimed to be the first to describe a recurrent translocation of EWS (22q12) and CREB1 (2q32.3) resulting in EWS-CREB1 fusion. They concluded that these cases may represent a gastrointestinal neuroectodermal tumor that express neuroectodermal markers and lacks melanocytic differentiation. However, the existence of rare cases of CCS of the gastrointestinal tract with EWS-ATF1 gene fusion that also lack melanocytic differentiation argue in favor of common histiogenesis between the two tumors [10]. In one study, translocation involving the gene EWSR1 was detected in 86% (Twelve cases) of the cases described as GNET. In the same study, the partner gene found to be ATF1 and was detected in 50% of the cases and CREB1 in 25% of the cases [1]. Two more studies have described rare EWSR1-CREB1 fusion in non-gastrointestinal CCS of soft tissue, however, these cases expressed melanocytic markers [20, 21]. In our case, FISH study for EWSR1 gene was performed recently after recognition of this entity and it was rearranged in keeping with the diagnosis of GNET.

Some authors regard this entity as CCS in gastrointestinal tract and explain the lack of melanocytic differentiation as being a variant of CCS especially after detecting EWSR1 rearrangement which is involved in CCS [16, 17]. Other authors considered that lack of osteoclast-like multinucleated giant cells argues against being a different entity from CCS [12, 14]. However, the recognition of the increasing number of these cases in the gastrointestinal tract associated with lack of melanocytic differentiation and worse clinical behavior compared to CCS of soft parts supports the notion of being a different entity [22]. A larger number of cases needs to be studied for better histological and molecular classification.

The differential diagnosis of GNET includes GIST, melanoma, CCS, MPNST and synovial sarcoma. GIST needs to be excluded first because of the location of the tumor and histologic features. GISTs feature spindle and/or epithelioid cells which makes the distinction from GNET difficult. However, GIST is not characterized by osteoclast-like multinucleated giant cells and it expresses CD117, DOG-1 and CD34 immunohistochemically. Clear cell sarcoma and melanoma need to be considered because of some shared histological features with GNET and S100 protein reactivity. The melanocytic markers in this situation are invaluable to rule out these diagnoses. Cytogenetic study for EWSR1 may not help to separate GNET from CCS but electron microscopy may have a role in revealing the melanocytic differentiation which is not present in GNET. MPNST can enter in the differential diagnosis because of similar histology (spindle and/or epithelioid cells) and S100 protein reactivity. However, MPNST usually doesn’t show strong and diffuse reactivity for S100 protein seen in GNET and it lacks the genetic translocation involving EWSR1 gene [11]. Monophasic synovial sarcoma can arise in the GI tract and the distinction from GNET can be difficult based on morphology alone. Synovial sarcoma is characterized by epithelial markers expression but S100 protein can be expressed. In difficult cases, the detection of SYT gene rearrangement t(X;18) can help in the distinction. An unusual finding of oncocytic cytoplasm has been recently described in GNET which raises the possibility of malignant granular cell tumor since both tumors express S100 protein and SOX10 immunoreactivity. Awareness of this finding in GNET may prompt the pathologist to avoid misdiagnosing it as malignant granular cell tumor and investigate EWSR1 gene status to confirm the diagnosis of GNET [9].

In conclusion, GNET is an extremely rare malignant tumor of the GI tract that can be mistaken for other non-epithelial GI tumors. Awareness of its existence and diagnostic criteria by the pathologist is necessary to avoid misdiagnosis, particularly as GIST, CCS or MPNST.

## Abbreviations

CCS: Clear cell sarcoma
CCSLTGT: Clear cell sarcoma-like tumor of the gastrointestinal tract
CT: Computed tomography
FISH: Fluorescein in situ hybridization
GI: Gastrointestinal tract
GIST: Gastrointestinal stromal tumor
GNET: Malignant gastrointestinal neuroectodermal tumor
MPNST: Malignant peripheral nerve sheath tumor
